# [Retracted] MicroRNA‑139‑5p inhibits cell viability, migration and invasion and suppresses tumor growth by targeting HDGF in non‑small cell lung cancer

**DOI:** 10.3892/ol.2023.14090

**Published:** 2023-10-09

**Authors:** Zuxiong Zhang, Weizhi Li, Damei Jiang, Chi Liu, Zhenghong Lai

Oncol Lett 19: 1806–1814, 2020; DOI: 10.3892/ol.2020.11296

Following the publication of this paper, it was drawn to the Editors’ attention by a concerned reader that, for the Transwell migration assay experiments shown in Fig. 5B on p. 1812, there were two instances of overlapping data panels, suggesting that data which were intended to show the results from differently performed experiments had been derived from the same original source. In addition, certain of the data shown in the invasion assay experiments in Fig. 5C subsequently appeared in a more expansive form in the following paper, published by different authors at different research institutes: Wang H, Niu X, Jiang H, Mao F, Zhong B, Jiang X and Fu G: Long non-coding RNA DLX6-AS1 facilitates bladder cancer progression through modulating miR-195-5p/VEGFA signaling pathway. Aging 12: 16021–16034, 2020.

Although the authors did respond to our query and attempted to explain the issues associated with this problematic figure, the Editor of *Oncology Letters* has decided that the paper should be retracted on account of an overall lack of confidence in the presented data. The Editor apologizes to the readership for any inconvenience caused.

